# Dissociation of the respiratory syncytial virus F protein-specific human IgG, IgA and IgM response

**DOI:** 10.1038/s41598-021-82893-y

**Published:** 2021-02-11

**Authors:** Kristina Borochova, Katarzyna Niespodziana, Margarete Focke-Tejkl, Gerhard Hofer, Walter Keller, Rudolf Valenta

**Affiliations:** 1 Department of Pathophysiology and Allergy Research, Division of Immunopathology, Center for Pathophysiology, Infectiology and Immunology, Medical University of Vienna, Vienna, Austria; 2 Institute of Molecular Biosciences, BioTechMed Graz, University of Graz, Graz, Austria; 3 NRC Institute of Immunology FMBA of Russia, Moscow, Russia; 4 Laboratory for Immunopathology, Department of Clinical Immunology and Allergy, Sechenov First Moscow State Medical University, Moscow, Russia; 5 Karl Landsteiner University of Health Sciences, Krems, Austria

**Keywords:** Immunology, Adaptive immunity, Humoral immunity, Antibodies

## Abstract

Human respiratory syncytial virus (RSV) is one of the most important causes of severe respiratory tract infections in early childhood. The only prophylactic protection is the neutralizing antibody, palivizumab, which targets a conformational epitope of the RSV fusion (F) protein. The F protein is generated as a F0 precursor containing two furin cleavage sites allowing excision of the P27 fragment and then gives rise to a fusion-competent version consisting of the N-terminal F2 subunit and the a C-terminal F1 subunits linked by two disulphide bonds. To investigate natural human F-specific antibody responses, F2 conferring the species-specificity of RSV, was expressed in *Escherichia coli*. Furthermore, the F0 protein, comprising both subunits F2 and F1, was expressed as palivizumab-reactive glycoprotein in baculovirus-infected insect cells. Six overlapping F2-derived peptides lacking secondary structure were synthesized. The analysis of IgG, IgA and IgM responses of adult subjects to native versions and denatured forms of F2 and F0 and to unfolded F2-derived peptides revealed that mainly non-conformational F epitopes, some of which represented cryptic epitopes which are not exposed on the proteins were recognized. Furthermore, we found a dissociation of IgG, IgA and IgM antibody responses to F epitopes with F2 being a major target for the F-specific IgM response. The scattered and dissociated immune response to F may explain why the natural RSV-specific antibody response is only partially protective underlining the need for vaccines focusing human antibody responses towards neutralizing RSV epitopes.

## Introduction

Respiratory syncytial virus (RSV) is the leading infectious cause of severe respiratory tract disease in children and is a major cause of mortality during infancy^1–3^. RSV can affect people of all ages and also represents a substantial health burden in adults over 65 years of age and in immune-compromised individuals^4–7^. Moreover, early-life RSV infection seems to predispose for the development of recurrent wheezing and asthma in childhood^8^. RSV is an enveloped, negative sense, single-stranded RNA virus which belongs to the *Pneumoviridae* family, the genus of *Orthopneumovirus* and the order *Mononegavirales*^9^. The virus was isolated first in 1955 from chimpanzees with respiratory illness and later also from infants suffering from bronchiolitis^10–12^. The RSV genome encodes 11 proteins among which the small hydrophobic (SH), the attachment (G) and the fusion (F) belong to the envelope proteins. RSV exists as a single serotype but is classified into two antigenic subgroups, RSV-A and RSV-B, which can be distinguished based on sequence variations in the attachment (G) protein and reactivity with monoclonal antibodies^13,14^. Strains from both antigenic subgroups can co-circulate at the same time of the year and are responsible for epidemics which show global seasonality^15–17^.

Despite the high burden of disease, no vaccine for RSV has been approved. Several promising vaccine candidates and RSV-specific treatment strategies are under development^18–20^. However, the only currently available specific therapy is a neutralizing monoclonal antibody for passive immunization, palivizumab (Synagis), which is used to prevent disease in high-risk infants^21^. Palivizumab was obtained by humanization of a RSV-neutralizing mouse monoclonal antibody specific for the fusion F protein^22,23^.

The F protein is essential for viral infectivity and binds to nucleolin which has been described as the cellular receptor for RSV^24^. The nucleotide and deduced amino acid sequence of the F protein have been determined and several studies have elucidated the structure of the F protein in its pre- and post-fusion conformation^25–30^. The F protein belongs to the family of class I viral fusion proteins and is responsible for the fusion of the viral envelope and cell membrane during infection^31^. The RSV F protein is generated as a F0 precursor containing two furin cleavage sites that allow excision of the P27 fragment in the trans-Golgi network to give rise to a fusion-competent version consisting of the N-terminal F2 subunit and the a C-terminal F1 subunits which are linked by two disulphide bonds. Much attention has been focused on the F1 subunit because it is responsible for fusion with the target cell membrane and harbours epitopes for several neutralizing monoclonal antibodies^27^. The F2 subunit has been less extensively studied although it contains epitopes which are recognized by neutralizing antibodies^30,32^. The F2 subunit is also of interest because it shows sequence variability among RSV strains from the two antigenic subgroups and especially varies between RSV strains infecting different species^33,34^. In this context it is of note that the F2 subunit has been identified as the major determinant of RSV host specificity^35^.

In this study the expression, purification and characterization of the F2 subunit containing P27 is reported. In addition, a series of overlapping peptides spanning the complete F2 subunit was produced. Furthermore, a recombinant palivizumab-reactive, soluble F protein with a mutated first furin cleavage site, deleted P27, linking F1 to F2 without transmembrane and cytoplasmic domains was expressed and purified to study the natural F-specific human antibody response regarding key isotypes (IgG, IgA and IgM). The results of our study reveal several novel aspects of the natural human antibody responses specific for the F protein of RSV.

## Results

### Strategy for the expression of recombinant F2-P27 and F0

**Figure 1:**
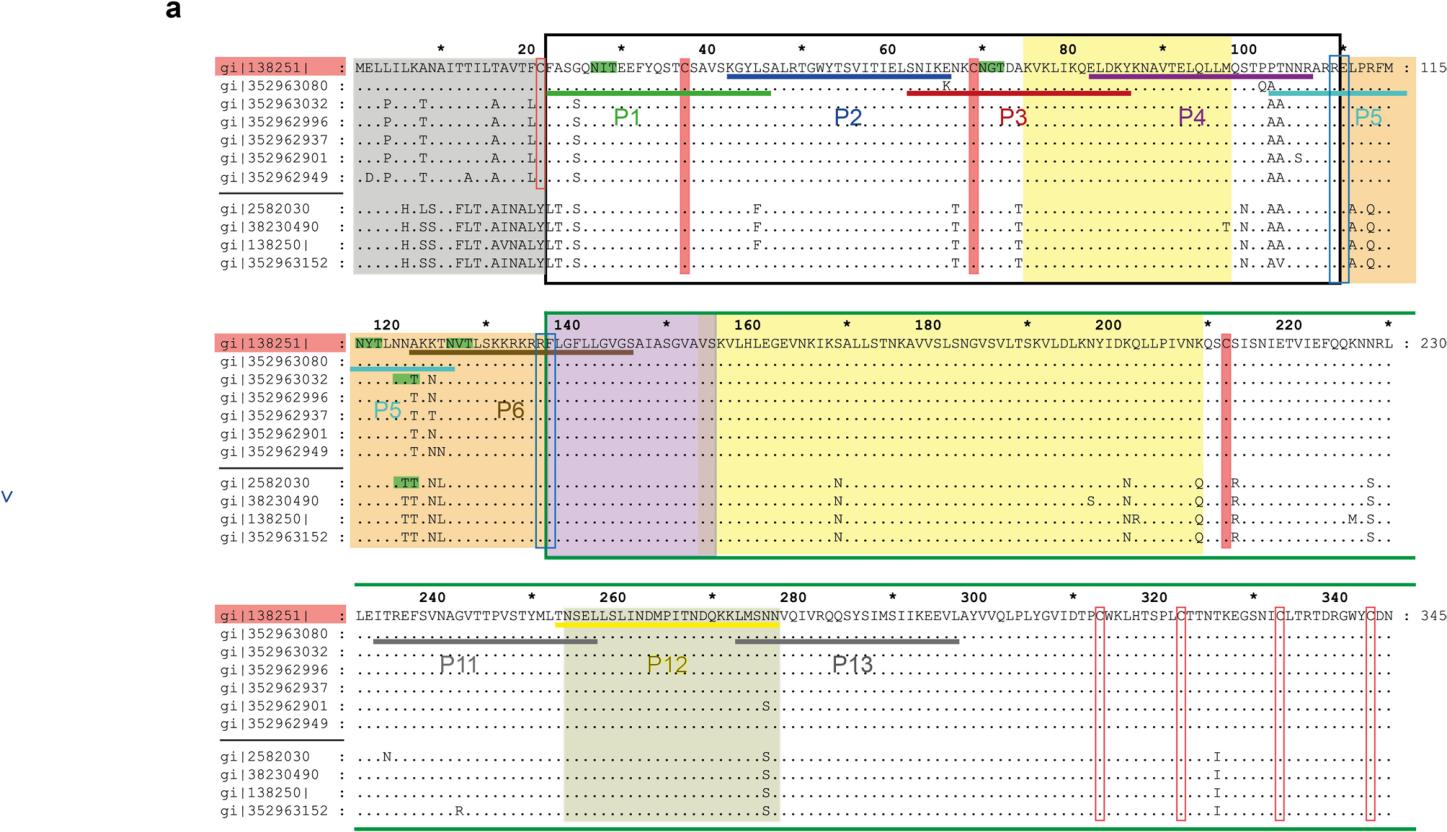

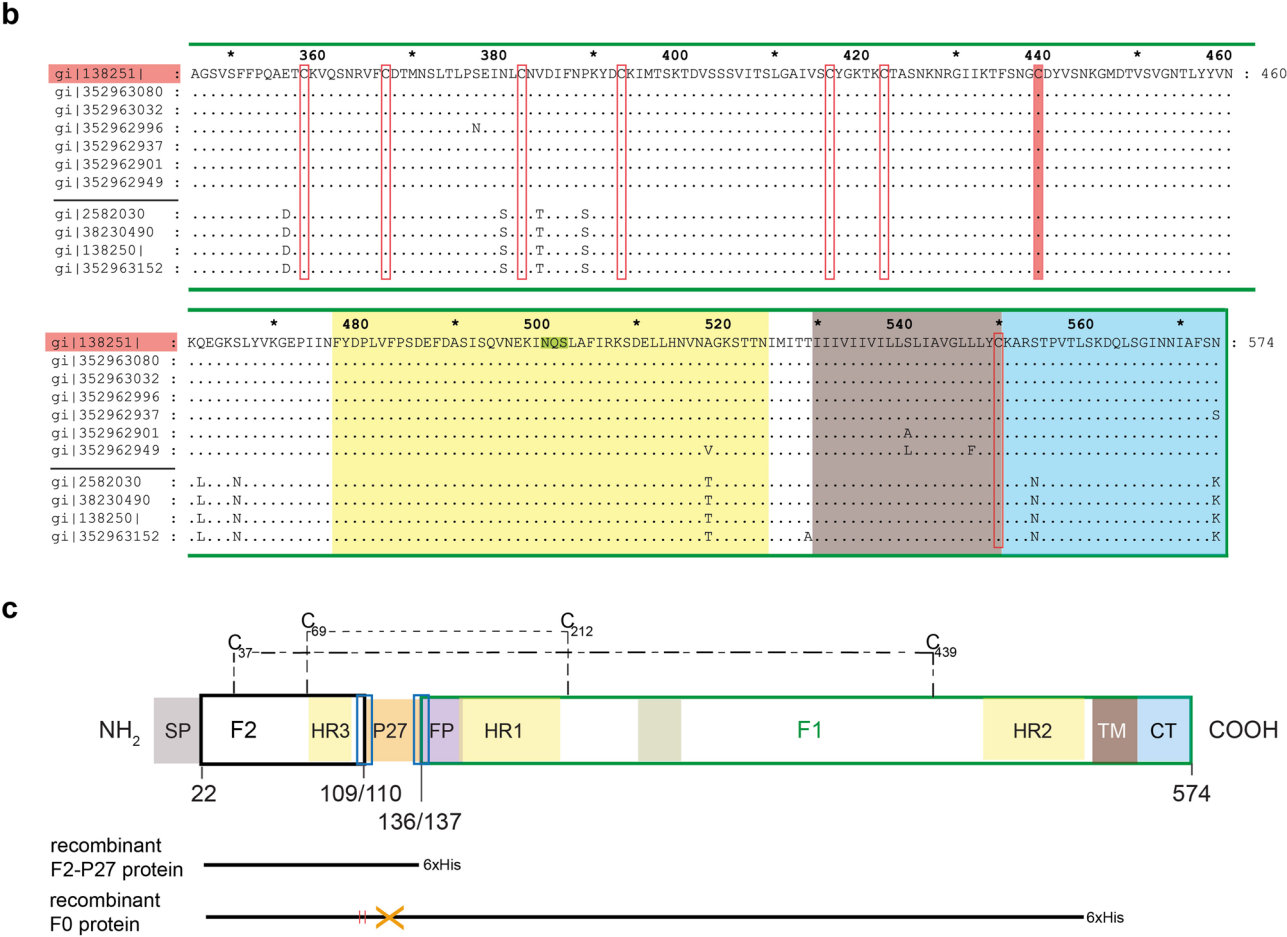
Illustration of A2 strain-derived recombinant F2-, F0-proteins and synthetic peptides. Alignment of the amino acid sequence of F0 from the A2 strain, subgroup A (top line), with the most variable strains from the two antigenic subgroups, A and B. Accession numbers for subgroups A and B (https://www.ncbi.nlm.nih.gov/) on the left margin are separated by a black horizontal line, amino acid numbers are indicated on top and on the right margin (**a**, **b**). Identical amino acids are indicated by dots, conserved cysteines are indicated by red boxes. Cysteines forming disulfide bonds between the F1 fragment (green box) and F2 fragment (black box) are indicated as filled red boxes whereas other cysteines are shown by open red boxes. The two furin cleavage sites (amino acid aa 109–110; aa 136–137) are indicated by blue boxes and predicted N-linked glycosylation sites are highlighted in green. The following parts are indicated: Signal peptide (grey box), heptad repeats HR1-3 (yellow boxes), cleaved peptide P27 (orange box), fusion peptide FP (purple box), transmembrane domain (brown box), cytoplasmic tail (blue box) and palivizumab binding site (khaki box). Synthetic peptides (P1, P2, P3, P4, P5, P6, P11, P12 and P13) are shown with horizontal bars. Schematic diagram of the complete fusion protein with disulfide bonds between the F2 and F1 fragment (**c**). Different parts are colored as in (**a**, **b**). Recombinant F2-P27 and F0 with C-terminal hexahistidine tails are indicated below.

For the expression of recombinant F2-P27 and F0 proteins we selected the A2 strain amino acid sequence (subgroup A: GenInfo Identifier: gi|138251 from https://www.ncbi.nlm.nih.gov/). The deduced amino acid sequence of complete F0 protein of A2-RSV strain has been aligned with the sequences of the most variable strains from the two antigenic subgroups, A and B in Fig. 1a, b. An extended version of the F2 subunit, containing the P27 fragment and both furin cleavage sites, termed F2-P27, was expressed in *E. coli*. The recombinant protein which was expressed as non-glycosylated protein was designed to include amino acids 22–136 (Fig. 1) and to contain a C-terminal hexahistidine tail to facilitate its purification by Nickel affinity chromatography.

Thus, the recombinant F2 protein was made to contain the heptad repeat region (HR3, K75-M97) (Fig. 1c) which is supposed to play an important role for the stability and conformation of the F protein^36^. Furthermore, our recombinant F2 protein contains the entire P27 fragment (F2-P27). As illustrated in the sequence alignment in Fig. 1, the recombinant F2-P27 is the most divergent segment regarding its amino acid sequence among the antigenic groups of human RSV strains^33^. Furthermore, recombinant F2-P27 contains the portion of the fusion protein which shows the lowest sequence conservation among RSV strains which are pathogenic in human and animals (e.g., cows) and accordingly was found to be responsible for host cell specificity of RSV^34,35^.

The construct for expression of recombinant F0 protein in insect cells was designed to contain a mutation of the first furin cleavage site I (RARR to QAQR) to prevent cleavage by furin proteases (Fig. 1). Furthermore, the P27 fragment with the second furin cleavage site II (amino acids 110–136, KKRKRR), the hydrophobic transmembrane anchor and the hydrophilic cytoplasmic tail (amino acids 525–574) were deleted. Recombinant F0 with the modification described above, comprising amino acids Q 26 to N 524 was expressed with a C-terminal hexahistidine tag as glycosylated protein in insect cells (Fig. 1c).

### Characterization of recombinant F2-P27

**Figure 2:**
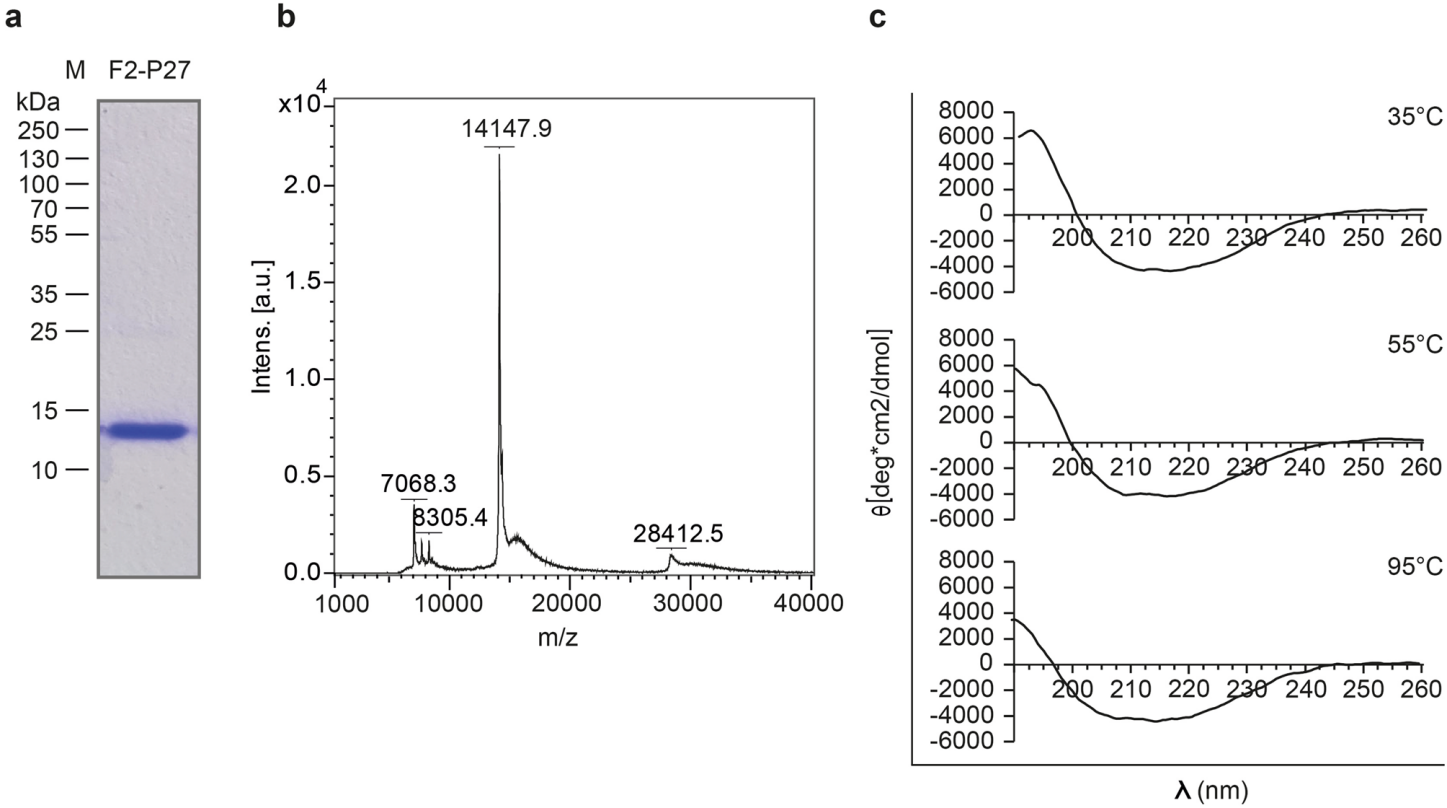
Characterization of recombinant F2-P27 protein. (**a**) Coomassie brilliant blue-stained SDS-PAGE showing purified F2-P27 subunit. Molecular weights (kDa) are indicated on the left margin. (**b**) Mass spectrometry of recombinant F2-P27 subunit. The mass/charge (m/z) ratios are shown on the x-axes, and the intensities are displayed on the y-axes in arbitrary units (a.u.). (**c**) Far-UV CD spectra of F2-P27 subunit at different temperatures (35°C, 55°C and 95°C). Mean residue ellipticities (θ) (y-axes) at given wavelengths (x-axes).

Recombinant F2-P27 was soluble in physiologic buffers and migrated at 14 kDa in SDS-PAGE (Fig. 2a). Matrix-assisted laser desorption/ionization time-of-flight analysis (MALDI-TOF) shows a main peak of 14,147.9 Da for F2-P27 which is in agreement with the predicted mass of 14,150 Da by ProtParam software for the recombinant F2-P27 protein with methionine and hexahistidine-tag (Fig. 2b).

**Figure 3:**
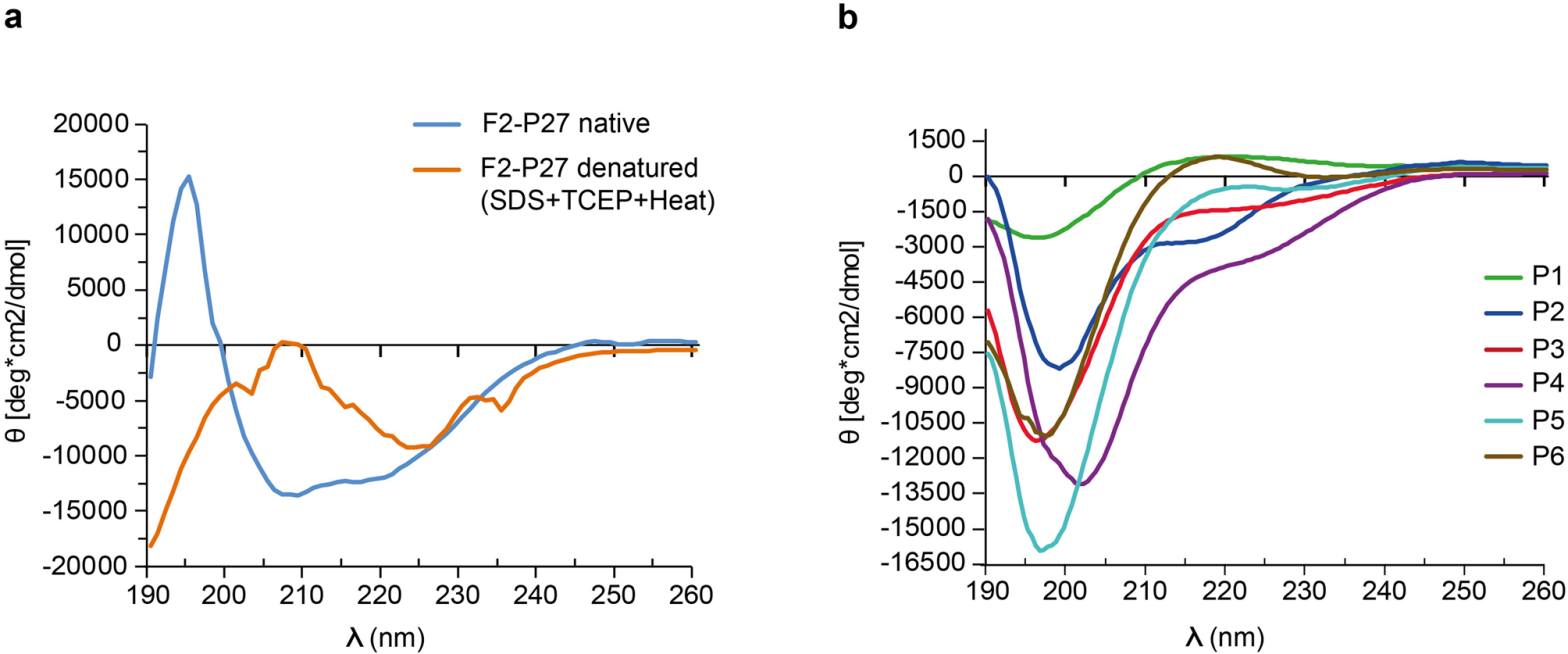
Circular dichroism analysis of recombinant F2-P27 protein subunit and F2-derived synthetic peptides. (**a**) Circular dichroism spectra of native (blue) or denatured (orange) F2-P27 subunit. (**b**) Circular dichroism spectra of F2-derived synthetic peptides (P1: green; P2: dark blue; P3: red; P4: purple; P5: cyan; P6: brown). Mean residue ellipticities (θ) (y-axes) at given wavelengths (x-axes) are shown.

The analysis by far-UV CD showed that the spectrum of recombinant F2-P27 protein was characterized by minima at 208–210 nm and at 218–220 nm consistent with a protein resembling a mix of alpha-helical and beta-sheet secondary structure. We also analyzed recombinant F2-P27 at different temperatures (Fig. 2c) and found that the protein exhibited a high thermal stability. Even at a temperature of 95 °C the protein seemed to preserve its secondary structure (Fig. 2c, lower part). However the addition of 15 mM of the reducing reagent TCEP, 0.46% SDS and pre-heating (95 °C) of the protein sample caused a structural transition to a predominant random coil structure with a negative peak starting at ~ 190 nm and a broad negative band at 220–230 nm (Fig. 3a).

### F2-derived synthetic peptides lack secondary structure

**Figure 4:**
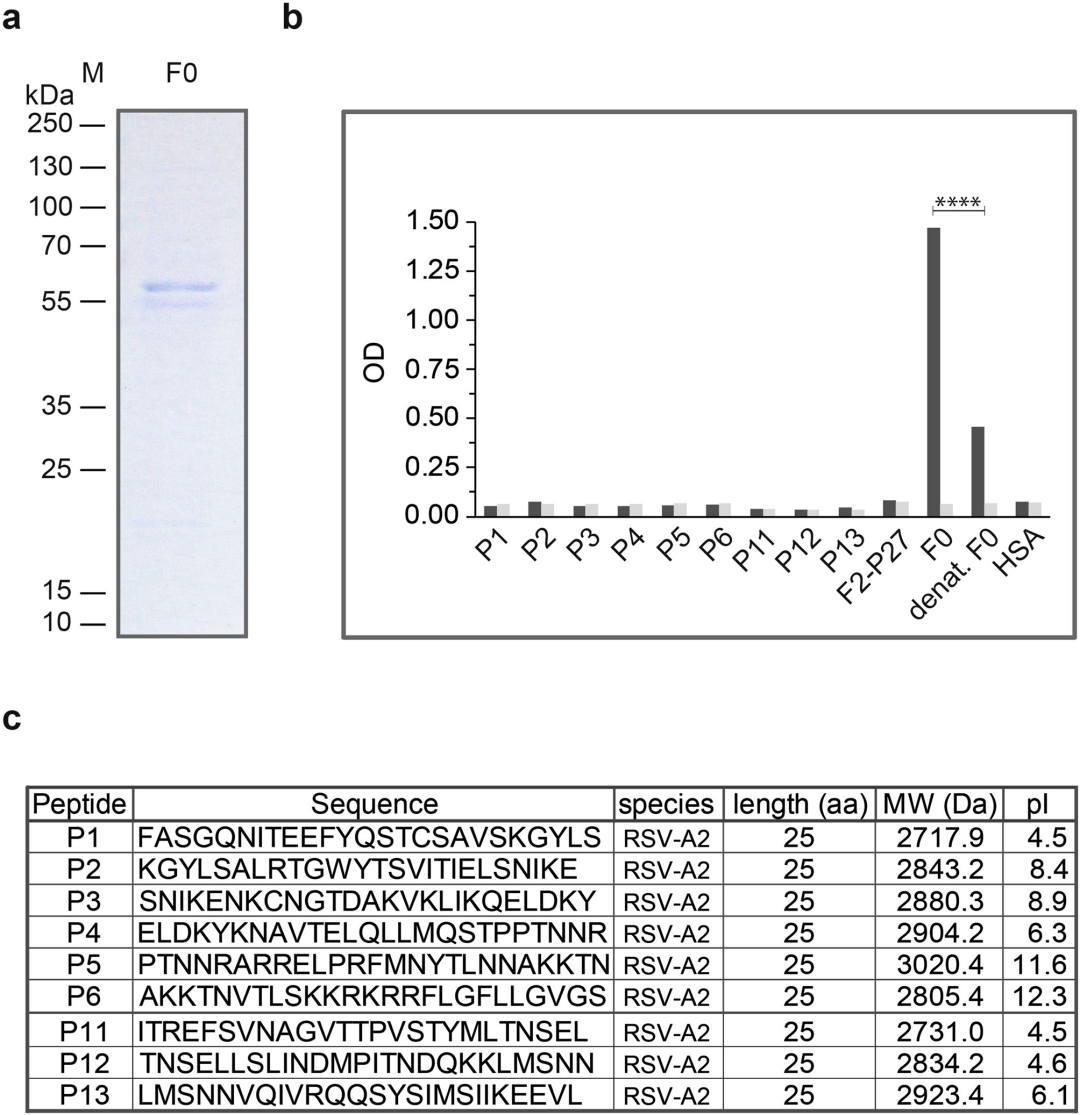
Characterization of recombinant F0 protein. (**a**) Coomassie brilliant blue-stained SDS-PAGE of recombinant F0 protein. Molecular weights (kDa) are indicated on the left margin. (**b**) Reactivity of palivizumab to native, denatured F0 and F0-derived peptides by ELISA. Optical density (OD) values (y-axis) correspond to bound human IgG antibodies, palivizumab (black bars) or buffer control (grey bars). Significant difference of palivizumab binding to native and denatured F0 is indicated (p < 0.0001; Mann–Whitney-U-test). (**c**) Characterization of synthetic peptides used (sequences, length, molecular weights, isoelectric points: pIs).

For the mapping of F2-specific antibody responses we synthesized six overlapping peptides of 25 amino acids length which spanned the F2-P27 sequence (Figs. 1a, 4). Figure 3b shows the CD spectra of the six overlapping peptides (P1–P6). They were characterized by minima around 195–202 nm consistent with the spectra of unfolded molecules. According to Kyte-Doolittle hydrophobicity and surface accessibility calculated by ExPASy Protscale, peptides P2 and P4 seemed to be hydrophobic and buried whereas peptides P3 and P6 displayed high surface accessibility (data not shown).

### Expression, purification and characterization of palivizumab-reactive recombinant F0

Recombinant soluble F0 protein could be expressed in baculovirus infected insect cells and purified by Nickel-affinity chromatography. Figure 4a shows the Coomassie-stained SDS-PAGE of recombinant F0 which appeared in the form of two bands of approximately 57 and 55 kDa corresponding to differently glycosylated versions of the protein. Both bands were found to be reactive with a monoclonal anti-His-tag antibody (data not shown) confirming their identity. The therapeutic human monoclonal antibody palivizumab which is derived from the mouse monoclonal antibody mAb 1129 requires the intact helix–loop–helix secondary structure of F0^22,23,37^. We therefore used palivizumab to investigate the presence and correct assembly of the palivizumab epitope on recombinant F0 by ELISA experiments (Fig. 4b). We found that palivizumab reacted strongly with recombinant F0 and that denaturation of recombinant F0 almost completely abolished binding of palvizumab (Fig. 4b). No binding of palivizumab to recombinant F2-P27, F2-derived peptides or to HSA was observed (negative controls, Fig. 4b,c). Palivizumab also did not bind to synthetic peptides (P11, P12, P13) comprising the amino acids which are part of the conformational epitope of palivizumab on F0 (Fig. 4b,c).

### F2- and F0-specific IgG antibodies from adult RSV-exposed subjects recognize non-conformational epitopes

**Figure 5:**
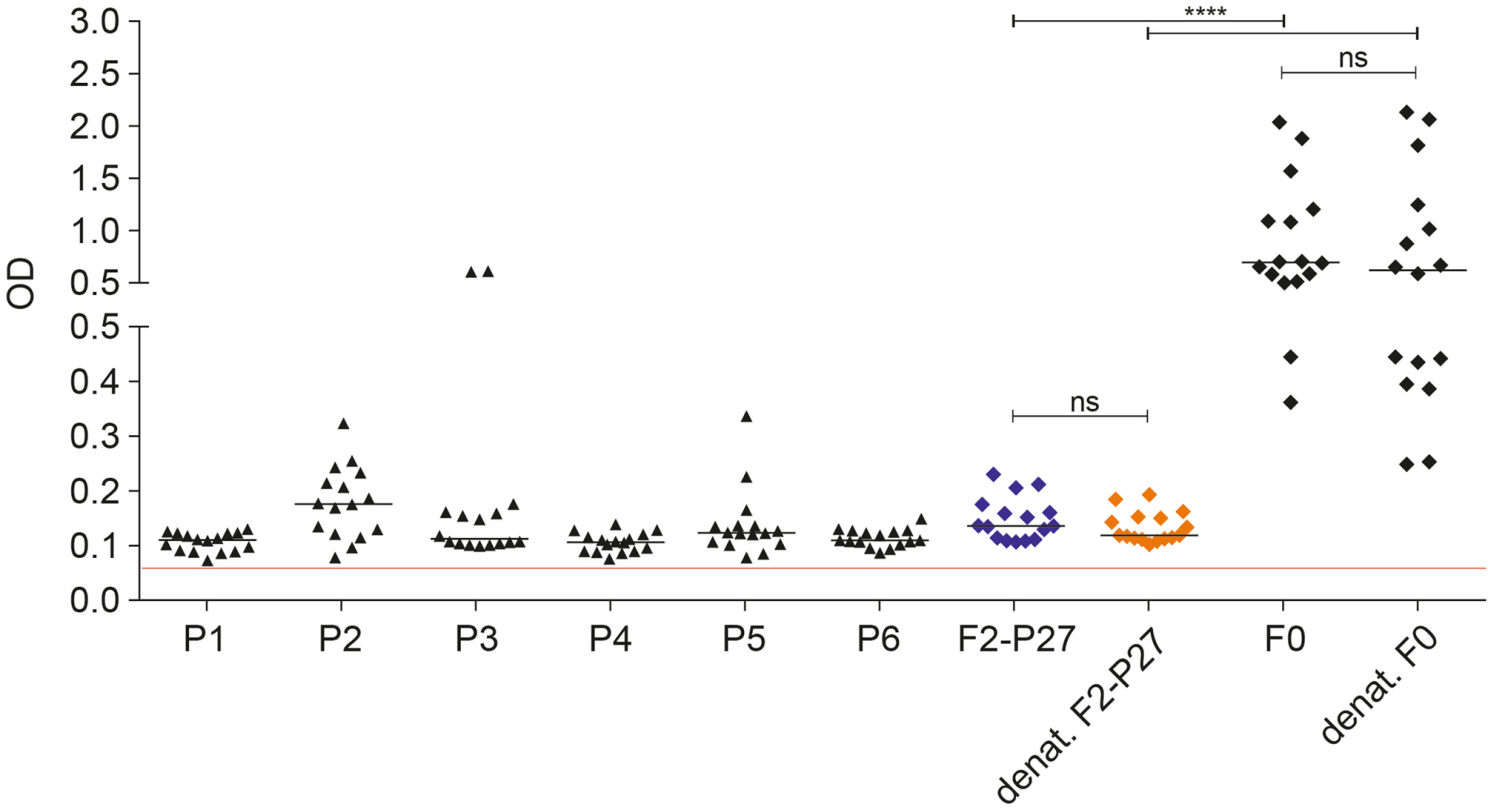
Effects of denaturation on human IgG responses to recombinant F2-P27 and F0. Shown are IgG levels (y-axis: optical density values) as scatter plots (16 adult individuals) specific for native (dark blue) and denatured (orange) recombinant F2-P27 subunit, native and denatured F0 and native F2-derived synthetic peptides (P1–P6) (x-axis). Horizontal lines within plots indicate median values. The cut-off (mean of buffer control plus three times standard deviation) is indicated by a red line. Significant differences of antibody responses are indicated: ns (not significant); *p < 0.05; **p < 0.001; ***p < 0.0001.

Sera from adult subjects were collected at the same time of the year (i.e., October–April) to reduce the possibility of variations of RSV-specific antibody responses due to seasonal differences in RSV exposure. Figure 5, shows the IgG reactivity of 16 subjects to recombinant native F2-P27, denatured F2-P27, recombinant native F0 and denatured F0 as well as to 6 unfolded peptides spanning the sequence of F2-P27. We found that there were no significant differences in the IgG responses to native and denatured F2-P27 and between native F0 and denatured F0 (Fig. 5). IgG antibody responses to F0 were significantly higher than to F2-P27 which represents a fragment comprising only approximately 25% of F0 (Fig. 5). For the 16 patients tested, peptides P2, P3 and P5 were frequently recognized by human IgG antibodies whereas peptides P1, P4 and P6 were less frequently reactive and IgG levels were lower (Fig. 5). In this context it should be noted that a large portion of P5 was part of the P27 region which upon intracellular cleavage of F0 is removed before it appears on the surface of infected cells. P3 contains several amino acids (i.e., SNIKENKC) which are reported to be part of the antigenic site Ø^30^. Since denatured F2-P27 and F0 showed similar IgG reactivity as native F2-P27 and F0 and unfolded F2-derived peptides were frequently recognized, it seems that IgG antibodies of adult subjects react mainly with non-conformational epitopes of F0.

### F2-P27 is a frequent target for IgM antibodies and contains cryptic epitopes

**Figure 6:**
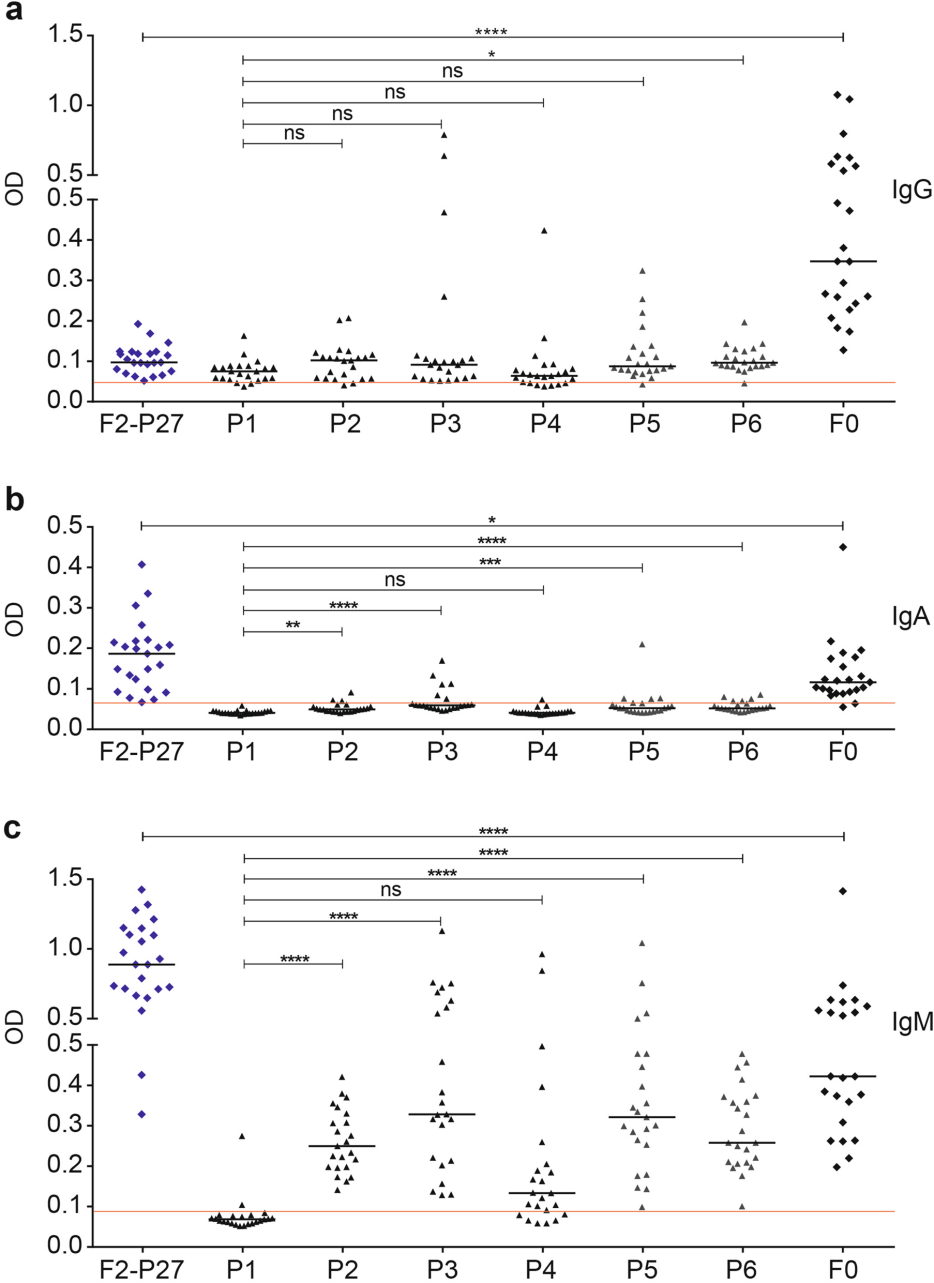
Human antibody responses to recombinant F2-P27, F0 and F2-derived synthetic peptides. Shown are IgG (**a**), IgA (**b**) and IgM (**c**) levels (y-axis: optical density values) in sera from 23 adult individuals to recombinant F2-P27, F0 and F2-derived synthetic peptides (P1–P6), (x-axis). Horizontal lines within scatter plots indicate median values. The cut-off (mean of buffer control plus three times standard deviation) is indicated by a red line. Significant differences of antibody responses are indicated: ns (not significant); *p < 0.05; **p < 0.001; ***p < 0.0001.

Next, we analyzed the IgG, IgA and IgM response of the adult subjects to native F2-P27, F0 and F2-derived peptides in more detail using sera from all 23 subjects. Regarding the IgG response we found again that the F0-specific IgG response was significantly higher than the F2-P27-specific IgG response (Fig. 6a). We noted that certain peptides such as P3, P5 and for one subject also P4 showed much higher IgG reactivity than complete recombinant F2-P27 (Fig. 6a). For example, subject 16 and 21 showed much stronger IgG reactivity to P3 than to F2-P27, subject 23 reacted stronger to P4 than to F2-P27 and subject 22 reacted stronger to P5 than to F2 (Supplemental Fig. 1) indicating the presence of cryptic contiguous epitopes in F2. The IgA response of the subjects was lower than the IgG response, in particular to F0. Interestingly, F2-P27 despite representing only 25% of F0 showed significantly stronger IgA reactivity than F0 (Fig. 6b). This surprising finding was even more pronounced for the IgM response. F2-P27 showed a significantly higher IgM reactivity than F0 and IgM reactivity to unfolded F2-derived peptides P2, P3, P5 and P6 was especially pronounced and significantly higher than IgM response to P1 (Fig. 6c). The IgM response was directed against P5 and P6 which overlap with the P27 region and P3 which contains amino acids of antigenic site Ø (Fig. 6c)^30^.

**Figure 7:**
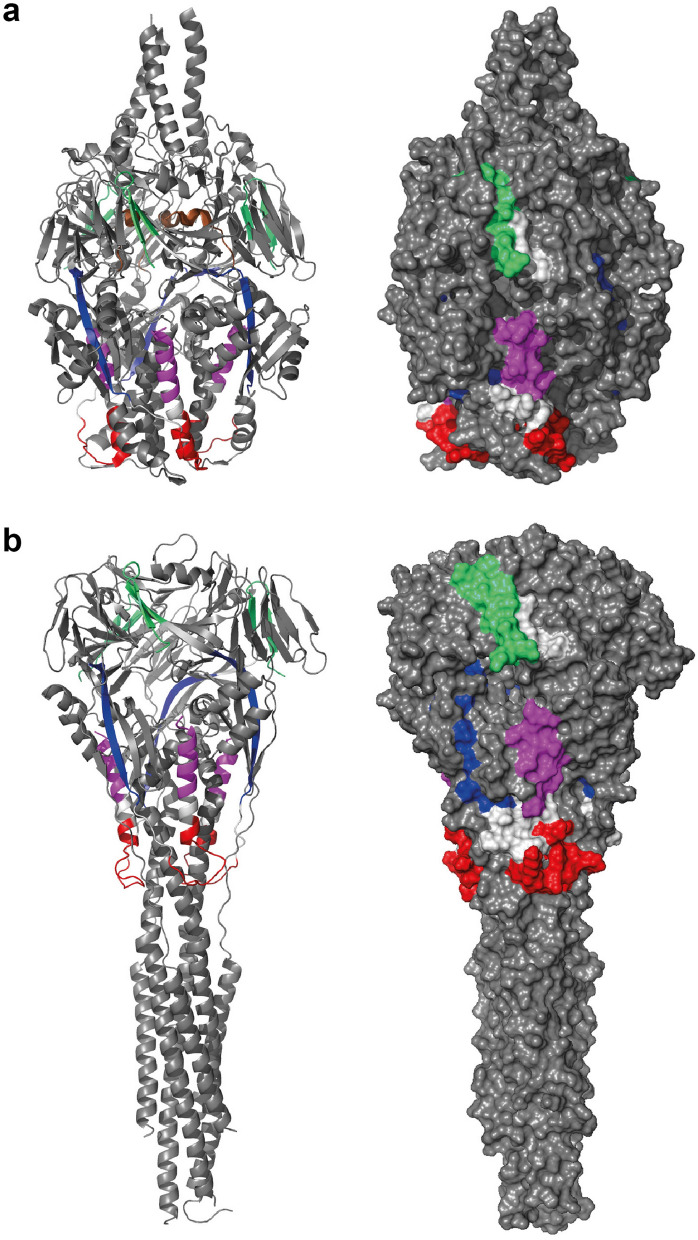
The surface exposure of the peptides in the folded forms of the RSV-F protein. (**a**) the pre-fusion F protein (PDB 3RRR) and (**b**) the post-fusion F protein (PDB 6APD) are shown in a cartoon-presentation (left panel) and as a surface presentation (right panel). Both forms exist as trimers with the three-fold axis aligned with the vertical axis. The peptides have been colored (P1 green, P2 blue, P3 red, P4 violet and P6 brown; the atomic coordinates for P5 and P6 are missing completely in the post-fusion F protein structure and only 10 residues of P6 have been determined in the pre-fusion F protein structure (brown α-helix in **a**). P1, P3 and P4 display several surface exposed regions, whereas P2 is mainly buried.

In order to localize peptides P1–P6 in the three-dimensional structure of the pre-fusion and post-fusion form of the F protein, the peptides were highlighted in the corresponding three-dimensional structures (Fig. 7a,b). Both forms exist as trimers with the three-fold axis aligned with the vertical axis. The atomic coordinates for P5 and P6 are missing completely in the post-fusion F protein structure and only ten residues of P6 have been determined in the pre-fusion F protein structure (brown α-helix in panel A). P1, P3 and P4 display several surface exposed regions, whereas P2 is mainly buried.

### Dissociation of the F protein-specific human IgG, IgA and IgM response

Supplemental Figs. 1–3 showed the detailed IgG, IgA and IgM responses for each of the subjects to each of the antigens in the form of a heatmap of specific antibody levels (i.e., F2-P27, F0, P1–P6). According to the heat map it appears as if the IgG, IgA and IgM response towards the different antigens is not synchronized. For example, peptide P3 is strongly recognized by IgG from subjects 16 and 21 but not from IgA or IgM of the same subjects. We therefore analyzed if the IgG, IgA and IgM responses against the individual antigens are correlated or not. Supplemental Figs. 4–6 show the correlations of IgG and IgA, of IgG and IgM and of IgA and IgM responses for each of the antigens. In fact, for most of the antigens no correlation of isotype responses was found which is summarized in Tables 1, 2 and 3 confirming that IgG, IgA and IgM responses are not synchronized but rather dissociated in adult subjects.

**Table 1:** Correlations between IgG and IgA levels to F2-P27, F0 and F2-derived peptides.

IgG	IgA	F2-P27	F0	P1	P2	P3	P4	P5	P6
F2-P27		r = − 0.209 p = 0.340							
F0			r = − 0.168 p = 0.444						
P1				r = 0.387 p = 0.068					
P2					r = 0.658 p = 0.001*				
P3						r = 0.351 p = 0.101			
P4							r = − 0.125 p = 0.570		
P5								r = − 0.004 p = 0.986	
P6									r = 0.015 p = 0.947

Spearman correlation r; *p values less than 0.05 were considered as significant.

**Table 2:** Correlations between IgG and IgM levels to F2-P27, F0 and F2-derived peptides.

IgG	IgM	F2-P27	F0	P1	P2	P3	P4	P5	P6
F2-P27		r = − 0.142 p = 0.519							
F0			r = − 0.419 p = 0.047*						
P1				r = 0.180 p = 0.414					
P2					r = 0.202 p = 0.356				
P3						r = 0.133 p = 0.544			
P4							r = − 0.098 p = 0.655		
P5								r = − 0.047 p = 0.833	
P6									r = 0.233 p = 0.284

Spearman correlation r; *p values less than 0.05 were considered as significant.

**Table 3:** Correlations between IgM and IgA levels to F2-P27, F0 and F2-derived peptides.

IgM	IgA	F2-P27	F0	P1	P2	P3	P4	P5	P6
F2-P27		r = − 0.159 p = 0.468							
F0			r = − 0.166 p = 0.450						
P1				r = − 0.025 p = 0.911					
P2					r = 0.435 p = 0.038*				
P3						r = 0.328 p = 0.126			
P4							r = 0.123 p = 0.576		
P5								r = 0.164 p = 0.454	
P6									r = 0.088 p = 0.692

Spearman correlation r; *p values less than 0.05 were considered as significant.

## Discussion

RSV infections are a major cause of respiratory illness in young children and infant mortality^1, 2^. The RSV F protein is important for the infectivity of the virus because it mediates both binding to the receptor on host cells and membrane fusion^24,25^. Importantly, the F protein is recognized by several virus-neutralizing antibodies, of which palivizumab has been registered for passive vaccination of high risk children^21,38^. We have prepared a set of defined recombinant proteins and synthetic peptides for studying the natural human isotype (IgG, IgA, IgM) response against the F protein. Our study is the first to report the expression and purification of a F2-P27 subunit assuming secondary structure as detected by CD. F2 is important because it contains binding sites of virus-neutralizing antibodies and is responsible for the species-specificity of RSV^30,35,38^. According to CD analysis, the recombinant F2-P27 protein represented a protein consisting of mixed alpha helical and beta-sheet secondary structure. However, it needs to be born in mind and is a possible limitation of our study that the recombinant F2-P27 was expressed in *E. coli* and therefore lacks post-translational modifications which may play a role in antibody binding. Furthermore, recombinant F2-P27 produced by us contains the P27 portion which is removed by proteolytic cleavage of the F0 precursor from the F2 subunit before it assembles with the F1 subunit in F.

In addition, we succeeded to express a recombinant F0 in insect cells which resembled the epitope recognized by palivizumab. Furthermore, a set of synthetic overlapping peptides, each containing 25 amino acids, spanning F2 and three peptides derived from the binding site of palivizumab were prepared. The first set of experiments performed with palivizumab demonstrated that the recombinant F0 contained the epitope recognized by palivizumab because denaturation of F0 results in an almost complete loss of its binding (Fig. 4c). Palivizumab also did not react with unfolded synthetic peptide P12 containing the contiguous (i.e., sequential) epitope comprising amino acids 254–277 which is part of the palivizumab epitope^22,39^. This finding is of note, because viral-like particles presenting this peptide were recognized by palivizumab and antibodies induced with the particles inhibited palivizumab and exhibited virus-neutralizing activity^40^.

When we analyzed natural IgG, IgA and IgM response of adult subjects against F2-P27, F2-derived peptides, F0 and the three F1-derived peptides, several novel findings were made. First of all, our results indicated that natural IgG antibodies were directed mainly against sequential, non-conformational epitopes of F. In fact, we found that there was no significant difference in IgG reactivity to native F2-P27 versus denatured F2-P27 and likewise, palivizumab-reactive F0 showed comparable IgG reactivity as denatured F0 which had lost palivizumab reactivity. In addition, we found that several unfolded F2-derived peptides (e.g., P2, P3, P5) were frequently recognized by human IgG antibodies. One of these peptides, P3 contains several amino acids which are part of the antigenic site Ø recognized by a RSV-neutralizing antibody^30^. However, it is quite likely that the P3-specific antibodies are not virus-neutralizing because a recent study demonstrated that the inclusion of this peptide in lipid core vaccine candidates induced a specific immune response but the induced antibodies had no virus-neutralizing effects^41^. Another interesting result was that P5 was frequently recognized by human IgG antibodies although it is part of the P27 region which is removed from the F0 protein upon its maturation in the host cell before it appears on cell surface and in the infectious virus. One possible explanation for this could be that F0 is released from damaged virus-infected cells and becomes immunogenic in its immature preform. This possibility is supported by another finding: in fact, several subjects showed much stronger IgG responses to F2-derived unfolded peptides containing only sequential epitopes than to the complete F2-P27 protein. This indicates that these peptides represent cryptic epitopes which only become immunogenic for example after proteolysis or after destruction of the intact protein/virus. Especially peptide P2, which was frequently recognized by IgM > IgG antibodies seems to represent a cryptic epitope because it was largely buried in the structure of the pre-fusion and post-fusion F structure (Fig. 7). This may indicate that RSV liberates fragments and/or particles of F which may act as a decoy to trap antibody responses as suggested also for the G protein^42^.

Our finding that much of the F-specific IgG response is directed sequential epitopes, some of which are cryptic, would also fit with results obtained in a study demonstrating the presence of IgG antibodies in humans which can discriminate between the pre- and post-fusion conformation of F in humans^43^. In fact, sequential epitopes, such as portions of antigenic site Ø (e.g., P3) may be accessible only in the pre-fusion but not in the post-fusion form of F or vice versa. However, it is a limitation of our study that we cannot say if the F protein produced by us, although reactive with palivizumab, assumes a correct fold because we could not compare it with the recombinant pre-fusion and post-fusion F proteins for which the three-dimensional structures have been determined^28–30^. In fact, the F protein produced by us is different from the crystallized post-fusion F protein which consists of a cleaved F2 and F1 portion subsequently assembled in mammalian cells^28^ whereas in our protein the F2 and F1 portion are connected within one protein and were expressed in insect cells. Furthermore, our F protein was different from the crystallized pre-fusion F protein which was obtained by co-expression with a monoclonal antibody that stabilizes it in the pre-fusion form^30^. We also did not have antibodies available for testing our F protein which would discriminate between the pre-fusion and post-fusion F protein^30^. Accordingly, we may have missed F protein-specific conformational epitopes which are present only on the reported pre-fusion and post-fusion F proteins^45^ but this does not affect our finding of sequential and cryptic F epitopes.

The comparison of F-specific IgG, IgA and IgM responses provided some more interesting results. While the IgG response to F0 was significantly higher than to F2-P27, the opposite was observed for IgM. In fact, F2-P27 reacted significantly stronger with human IgM than F0 and as observed for IgG, IgM antibodies frequently reacted with unfolded peptide epitopes. An in depth analysis of the F2-P27-specific IgM response in children with different course of disease (mild versus severe) in future studies will be interesting to find out if high levels of F2-P27-specific IgM may be associated with more severe disease because of possible complement activation^44^.

In agreement with an earlier study, which reported low RSV-specific IgA responses in individuals which were experimentally infected with RSV we also found a low F-specific IgA response both against the recombinant proteins and peptides^45^. Using serum samples obtained from children who had experienced virus-triggered exacerbations of wheeze and were PCR positive for RSV we found increases of F0 and especially G-specific IgG responses approximately ten weeks after the wheezing exacerbation suggesting infection by RSV as a possible trigger factor^46^.

Finally, we analyzed the correlation of F and F-derived peptide-specific isotype responses. We found that IgG, IgA and IgM responses were dissociated in terms of magnitude and epitope recognition. Thus, it seems that the F-specific isotype responses is scattered which is different from certain other viral infections. For example, the natural rhinovirus (RV)-specific IgG, IgA and IgM was highly synchronized and directed against a N-terminal peptide of the VP1 coat protein^47^. This finding actually allowed to establish serological tests based on micro-arrayed VP1-derived peptides from different RV-strains for robust monitoring of RV-specific antibody responses for the diagnosis of RV-induced asthma exacerbations^48–50^.

Likewise, it was found that the natural HIV-specific antibody response was directed against distinct peptides of gp120 and gp41 which seem to be conserved among different HIV clades^51,52^.

In contrast, the RSV-specific isotype response was scattered similar as the allergen-specific IgE and IgG response in allergic subjects where most of the allergen-specific IgG antibodies seem to be directed against epitopes which are different from those recognized by IgE^53^. Accordingly, new types of allergy vaccines used for allergen-specific immunotherapy are designed to focus allergen-specific IgG responses against IgE epitope-containing regions to induce a protective immune response^54–56^.

Therefore, our data obtained for RSV support currently pursued strategies which suggest obtaining novel generations of improved RSV vaccines by engineering immunogens so that they can focus antibodies against neutralizing epitopes on RSV^18,19,26^.

## Materials and methods

All experiments were performed in accordance with relevant guidelines and regulations and all experimental protocols were approved. (https://www.meduniwien.ac.at/web/en/rechtliches/good-scientific-practice/).

### Expression in *E. coli *and purification of recombinant F2-P27 subunit

The amino acid sequence of the extended F2 subunit containing the P27 fragment and both furin cleavage sites was retrieved from UniProtKB/Swiss-Prot-Expasy: full Fusion Glycoprotein, FUS_HRSV (accession number: P03420.1) (Fig. 1). The cDNA was codon-optimized for the expression in *E. coli*, synthesized with the addition of a DNA coding for a hexahistidine tag at the 3′ end (Genscript, Piscataway, NJ, USA) and inserted into the 5′ NdeI and 3′ XhoI restriction sites of the pET-27b ( +) vector (ATG: biosynthetics, Merzhausen, Germany). Correct insertion of the F2 construct was confirmed by NdeI/XhoI restriction analysis of midi-prep plasmid DNA (Promega, Madison, WI, USA) followed by DNA sequencing (Eurofins MWG Operon, Ebersberg, Germany). Recombinant F2-P27 was expressed in BL21-Gold (DE3) *E. coli* cells (Agilent Technologies, USA) at 37 °C at 180 rpm overnight in the presence of 1 mM IPTG (isopropyl-b-d-thiogalactopyranoside). Recombinant F2-P27 protein was purified by affinity chromatography using Hi60 Ni–NTA resin (Clontech Laboratories, Inc.; Takara Biotechnology; Fitchburg, Wisconsin, US) under denaturing conditions (Qiagen, Hilden, Germany). Recombinant F2-P27 was enriched in inclusion bodies and purified as described by Gallerano et al*.*^57^. Pure protein fractions were pooled together and dialysed in a Slide-A-Lyzer dialysis cassette, cut-off of 3.5 kDa (Thermo Scientific, Pierce Protein Biology Products, Rockford, USA) against the storage buffer (10 mM acetic acid/natrium acetate, 0.15 M NaCl, pH 5 or ddH_2_O). His-tagged F2-P27 yielded 2.5 mg/L culture, of a more than 95% pure recombinant protein, which was soluble in physiologic buffers and migrated at 14 kDa in SDS-PAGE (Fig. 2a).

### Matrix-assisted laser desorption and ionization time-of-flight (MALDI-TOF) mass spectrometry of recombinant F2-P27

Purified recombinant F2-P27 protein was analysed by a MALDI-TOF mass spectrometer (Microflex, MALDI-TOF, Bruker Daltonics, Billerica, MA) using a SA (sinapinic acid dissolved in 50% acetonitrile, 0.1% trifluoroacetic acid) matrix solution. For sample preparation a 1:1 mixture of protein (0.2 mg/ml) and matrix solution was applied onto a target and air dried. Acquired spectra were analysed with the Bruker Daltonics FlexAnalysis software (Bruker Daltonics). Figure 2b, shows a main peak of 14,147.9 Da which is in agreement with the predicted mass of 14,150 Da by ProtParam software for the recombinant F2-P27 protein with methionine and hexahistidine-tag. The small and flat hill in the range of m/z 15,000 may have originated from protein- metal ion adduct formation^58^. The small peak at 28,412.5 Da may result from a F2-P27 dimer, since Matthews et al*.* showed that the heptad repeat 3 (HR3, 53–100 aa) region in the F2 subunit is able to form dimeric helices consistent with the observation of dimer formation via DLS and SDS-PAGE (data not shown)^59^. The peaks with low signal intensities and lower mass to charge values (7,068.3 Da, 8,305.4 Da) may correspond to the M2H + species of the protein.

### Peptide synthesis and purification

Overlapping peptides with a length of 25 amino acids and an overlap of 5 aa were produced by solid phase synthesis, using the same methods, resins and instruments as described by Borochova et al*.* 2020. Purification and confirmation of correct peptide identity was also performed as described previously^46^.

### Expression in baculovirus-infected insect cells and purification of recombinant F0

The amino acid sequence of the complete fusion envelope protein was retrieved from UniProtKB/Swiss-Prot-Expasy: full fusion glycoprotein F0 (accession number: P03420.1). The cDNA coding for the extracellular domain of F0 protein (amino acids Q 26 to N 524) was modified as follows: A mutation was introduced in the first furin cleavage site I (RARR–QAQR) to prevent cleavage by intracellular proteases. Furthermore, the P27 fragment with the second furin cleavage site II (amino acids 110–136, KKRKRR), the hydrophobic transmembrane anchor and the hydrophilic cytoplasmatic tail (amino acids 525–574) were deleted whereas a DNA coding for a C-terminal hexahistidine tag was added. This DNA construct was codon-optimized for insect cell expression, was synthesized (ATG: biosynthetics, Merzhausen, Germany) and cloned into the BamHI/SmaI sites of the pTM1 vector, harbouring the baculoviral polyhedron promoter sequence, similar to the protocol described by Pahr et al*.*^60^. The construct was confirmed by BamHI/SmaI restriction analysis of midi-prep plasmid DNA (Promega, Madison, WI, USA) followed by DNA sequencing (VBC-Biotech Service, Vienna, Austria). Baculovirus amplification was performed in Spodoptera frugiperda (Sf9) insect cell line, whereas expression was performed in 20 ml Trichoplusia ni (High Five) insect cell cultures at 27 °C for 3 days, yielding 0.3 mg/20 ml culture (F0). Sf9 and High Five cells were obtained from Life Technologies (Carlsbad, CA, US).

Recombinant F0 protein was purified under denaturing conditions from insect cells as described for recombinant F2-P27. After Nickel-affinity chromatography, fractions containing pure F0 were pooled and dialysed in Spectra/Por dialysis membrane tubes, cut-off of 12–14 kDa against the storage buffer (0.2 M NaCl, 10 mM Tris, 0.1 M NaH_2_PO_4_, pH 6.5). Identity and purity of recombinant F0 and F2-P27 were evaluated by 12% Coomassie-stained SDS-PAGE followed by immunoblotting using 1:1000 diluted monoclonal mouse anti-His-epitope tag antibody (Dianova, Hamburg, Germany). Bound antibodies were detected with 1:1000 diluted alkaline phosphatase-coupled rat anti-mouse IgG antibodies (BD Bioscience/Pharmingen, Heidelberg, Germany). For determination of correct assembly of the palivizumab epitope in F0, palivizumab (Synagis, Abbvie, Maidenhead, UK) was used in ELISA experiments using a 1:5000 dilution of HRP anti-human-IgG antibodies (BD Pharmingen, San Diego, CA, USA). Protein concentrations were determined in micro-plate format by BCA Protein Assay Kit (Pierce, Rockford, IL, US). The pre-stained protein molecular weight marker (PageRuler prestained Protein Ladder Plus, Fermentas, St Leon-Rot, Germany) was used as a standard in SDS-PAGE.

### Circular dichroism spectroscopy

Circular dichroism (CD) spectroscopy was recorded at 25 °C using a Jasco J-180 spectropolarimeter (Japan Spectroscopic Co., Tokyo, Japan) using a high transparency 1 mm light path quartz cuvette. The CD spectra were measured at a F2-P27 protein concentration of 0.1 mg/ml and peptide concentrations from 0.05–0.5 mg/ml (P1 0.05 mg/ml, P2 0.3 mg/ml, P3 0.5 mg/ml, P4 0.5 mg/ml, P5 0.3 mg/ml, P6 0.5 mg/ml) in ddH_2_O. Each spectrum represented an average of 5 scans collected from 190 to 260 nm, with a resolution of 0.5 nm and at a rate of 50 nm/min. The final spectra were noise-reduced using the instrument algorithm and baseline corrected by subtracting the spectrum of a buffer blank (ddH_2_O) obtained under identical conditions. Results were expressed as the mean residue ellipticity [θ] (Deg cm^2^/dg), at a given wavelength. Temperature scans of recombinant F2-P27 subunit were performed according to a step-scan procedure, where the sample was heated stepwise at 2 °C/min from 25 to 95 °C and finally cooled down to a starting point at the same rate. Three spectra were recorded every 5 °C, and averaged. The conformational state of the F2-P27 protein was calculated using the secondary structure estimation algorithm CDSSTR with reference set number 4, optimized for the wavelength range 190–260 nm as well as algorithms SELCON3 and CONTIN using DichroWeb (http://dichroweb.cryst.bbk.ac.uk/html/home.shtml). Results from the CD measurements were also compared to the sequence-based predictions of the protein secondary structure performed by PsiPred (bioinf.cs.ucl.ac.uk/psipred).

CD spectrometry of the F2-P27 subunit under denaturing conditions was performed under the same instrumental settings. Native F2-P27 protein sample (10 mM acetic acid/natrium acetate, 0.15 M NaCl, pH 5) was measured and baseline corrected as described above and compared with a thermally denatured and reduced F2-P27 sample (10 mM acetic acid/natrium Acetate, 0.15 M NaCl, pH 5, 0.46% sodium dodecyl sulfate (SDS), 15 mM tris(2-carboxyethyl) phosphine hydrochloride (TCEP)) which had been heated up to 95 °C for 5 min before measurement. Baseline correction was carried out using the respective buffers used for the proteins. Data were analysed and plotted using Microsoft Excel.

### Enzyme-Linked Immunosorbent Assay (ELISA) experiments

Stored residual serum samples, which had been collected during a period of the year when RSV infections are common (i.e., October to April) were pseudo-anonymized and analyzed with approval from the ethics committee of the Medical University of Vienna (EK1641-2014). Informed written consent was obtained from all subjects. Samples were from healthy adult subjects (n = 23; males/females: 7/16 age range: 21–58; mean age: 32.8 years). Please note that for the experiments, described in Fig. 5, only 16 subjects were available and analyzed. PCR testing for RSV or other respiratory viruses was not performed in the subjects when serum samples were obtained.

IgG, IgM and IgA reactivity of human sera were determined. Antigens (i.e., synthetic peptides, native F2-P27, heat-denatured and reduced F2-P27, native and heat-denatured-reduced F0) were coated at a concentration of 2 µg/ml overnight at 4°C onto ELISA plates (Microplate, 96 well, PS, half area, clear and binding; Greiner bio-one, Germany). The concentration of 2 µg/ml was established in pilot experiment to ensure excess of antigen. Coating of denatured and reduced proteins was carried out by using coating buffer (0.05 M carbonate-bicarbonate, pH 9.6, 0.46% w/v sodium dodecyl sulfate (SDS), 15 mM tris (2-carboxyethyl) phosphine hydrochloride (TCEP)) overnight (ON). Denatured protein samples were pre-heated 95 °C for 10 min before coating. For native protein samples and peptides, a coating buffer without SDS and TCEP was used (0.05 M sodium-bicarbonate, pH 9.6). After incubation, plates were washed two times with washing buffer (PBS + 0.05% Tween 20) and blocked with 2% w/v BSA (bovine serum albumin) solution (PBS + 0.05% Tween 20 and 2% w/v BSA) for five hours at room temperature (RT). Following the removal of blocking solution, a 1:50 serum dilution, in dilution buffer (PBS + 0.05% Tween 20 and 0.5% w/v BSA) was added or dilution buffer only (control wells). Plates were incubated ON at 4°C and on the next day washed five times with washing buffer and incubated at RT with appropriate antibody-dilution. Bound IgG antibodies were detected with horseradish peroxidase (HRP)-conjugated anti-human-IgG antibodies, diluted in dilution buffer 1:5000 (HRP anti-human IgG; cat: 55788; BD Pharmingen, San Diego, CA, USA), after one hour incubation time at RT and a five times washing step.

IgA and IgM binding was determined with monoclonal mouse anti-human IgA or IgM antibodies with a dilution factor of 1:1000 (IgA antibody cat.: 555886, clone: G18-1; IgM antibody cat: 555856, clone: JDC-15; BD Pharmingen, San Diego, CA, USA). IgA or IgM antibody dilutions were incubated on plates for two hours at RT. After washing, the plates five times with washing buffer a secondary incubation with the HRP-conjugated sheep anti-mouse IgG antibody (NA931; GE Healthcare UK Limited, Buckinghamshire, UK), diluted 1:2000 in dilution buffer, was carried out for one hour at RT. Following the final five times washing step, the colour reaction was started by adding 50 µl/well of substrate solution as described previously^46^. The optical density (OD) values relate to the levels of antigen-specific antibodies and were measured at 405 and 492 nm on Perkin Elmer EnSpire 2300 ELISA reader device^46^. Buffer controls, without addition of serum were also measured on each plate to calculate cut-off levels. All determinations were performed in duplicates, with a variation of less than 5%, and results were expressed as normalized mean values. Plate to plate normalization was accomplished by including a control serum with established antigen-specific antibody levels on each of the plates^61^.

### Localization of peptides on the three-dimensional structures of pre- and post-fusion F

Cartoon and surface depictions were created with PyMOL (PyMOL Molecular Graphics System, Version 2.0 Schrödinger, LLC). The PDB structures used were 6APD for the pre-fusion form and 6RRR for the post-fusion form.

### Statistical analysis and sequence comparison

Statistical analysis was performed using GraphPad Prism 6 Software. Antibody responses towards the six peptides were analysed by One-way ANOVA and Kruskal–Wallis test. For comparison of antibody responses to the two proteins (F2-P27, F0), Mann–Whitney-U-test was performed. A p value < 0.05 was considered statistically significant (p < 0.05 = *). The correlations between antigen-specific IgG, IgA and IgM levels were calculated by XY Correlation option using the nonparametric, two tailed Spearman correlation method. p values below 0.05 were considered as significant. Difference in palivizumab binding between denatured and native recombinant F0 protein was determined by Mann–Whitney-U-test. p value below 0.05 was considered as significant, p < 0.0001 = ****). For sequence alignment Clustal Omega-multiple sequence alignment program was used (https://www.ebi.ac.uk/Tools/msa/clustalo/).

## Supplementary Information

Supplementary Information.

## Data Availability

The dataset generated during and/or analyzed during the current study are available from the corresponding author on reasonable request.
