# Supplementary material for: Dissociation of the respiratory syncytial virus F protein-specific human IgG, IgA and IgM response
Source: Sci Rep. 2021 Feb 11;11:3551. doi: 10.1038/s41598-021-82893-y (PMC7878790; doi:10.1038/s41598-021-82893-y)

**Supplemental materials:**

**Dissociation of the respiratory syncytial virus F protein-specific human IgG, IgA and IgM response**

Kristina Borochova^1^, Katarzyna Niespodziana^1^, Margarete Focke-Tejkl^1^, Gerhard Hofer^2^, Walter Keller^2^, Rudolf Valenta^1,3,4,5*^

^1^Department of Pathophysiology and Allergy Research, Division of Immunopathology, Center for Pathophysiology, Infectiology and Immunology, Medical University of Vienna, Vienna, Austria.

^2^Institute of Molecular Biosciences, BioTechMed Graz, University of Graz, Graz, Austria.

^3^NRC Institute of Immunology FMBA of Russia, Moscow, Russia.

^4^Laboratory for Immunopathology, Department of Clinical Immunology and Allergy, Sechenov First Moscow State Medical University, Moscow, Russia.

^5^Karl Landsteiner University of Health Sciences, Krems, Austria.

* Corresponding author

E-mail: [rudolf.valenta@meduniwien.ac.at](mailto:rudolf.valenta@meduniwien.ac.at)

Phone: +43 1 40400 51080

Fax: +43 1 40400 51300

E-Mail address of the first author: [kristina.borochova@meduniwien.ac.at](mailto:kristina.borochova@meduniwien.ac.at)

**Keywords:** Respiratory syncytial virus (RSV), fusion protein, recombinant proteins, F2 subunit, epitope, antibody response.

**Acknowledgements**

This work was funded by the Austrian Science Fund (FWF), projects P29398 and the PhD program “Inflammation and Immunity”, IAI, the Medical University of Vienna and by Viravaxx, Vienna, Austria.

**Author Contributions:**

KB designed and performed experiments, analysed data, prepared the figures, wrote and read manuscript; KN: analysed data, supervised experiments, read manuscript; MFT: analysed data, read manuscript; GH: analysed data, prepared one figure, read manuscript; WK: analysed data, read manuscript; RV: designed and supervised experiments, analysed data, wrote and read manuscript. All authors reviewed and approved the manuscript.

**Statement regarding patient samples used in this study:**

Serum samples from adult individuals used in this study were pseudo-anonymized and analyzed with approval from the ethics committee of the Medical University of Vienna (EK1641-2014). Informed written consent was obtained from all subjects.

**Conflicts of interests:**

Rudolf Valenta has received research grants from Viravaxx, Vienna, Austria and serves as a consultant for this company. The other authors have no conflicts of interest to report.

**Figure legends:**

**Supplemental Figure 1.** Heat map of IgG antibody responses (OD values corresponding to bound antibodies) of 23 adult individuals (#1-23) to recombinant F2-P27, F0 and F2-derived synthetic peptides. Colors indicating different antibody levels are shown in the insert.

**Supplemental Figure 2.** Heat map of IgA antibody responses (OD values corresponding to bound antibodies) of 23 adult individuals (#1-23) to recombinant F2-P27, F0 and F2-derived synthetic peptides. Colors indicating different antibody levels are shown in the insert.

**Supplemental Figure 3.** Heat map of IgM antibody responses (OD values corresponding to bound antibodies) of 23 adult individuals (#1-23) to recombinant F2-P27, F0 and F2-derived synthetic peptides. Colors indicating different antibody levels are shown in the insert.

**Supplemental Figure 4.** Correlations of specific IgG levels (x-axis) and IgA levels (y-axis) specific for F2-P27, F0, P1, P2, P3, P4, P5 and P6 measured in sera from 23 adult individuals in individual scatter plots with r (Spearman correlation coefficient) and P values.

**Supplemental Figure 5.** Correlations of specific IgG levels (x-axis) and IgM levels (y-axis) specific for F2-P27, F0, P1, P2, P3, P4, P5 and P6 measured in sera from 23 adult individuals in individual scatter plots with r (Spearman correlation coefficient) and P values.

**Supplemental Figure 6.** Correlations of specific IgM levels (x-axis) and IgA levels (y-axis) specific for F2-P27, F0, P1, P2, P3, P4, P5 and P6 measured in sera from 23 adult individuals in individual scatter plots with r (Spearman correlation coefficient) and P values.


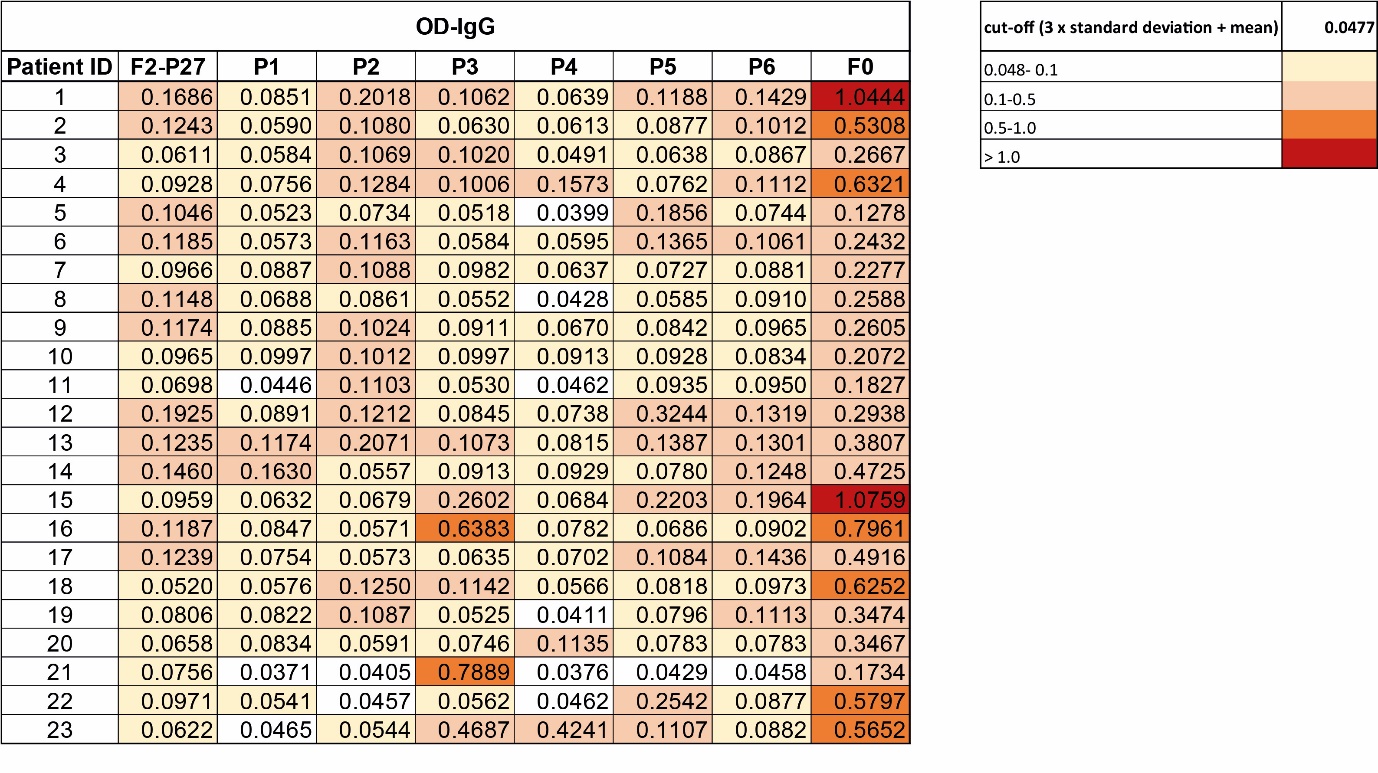


Supplemental Figure 1


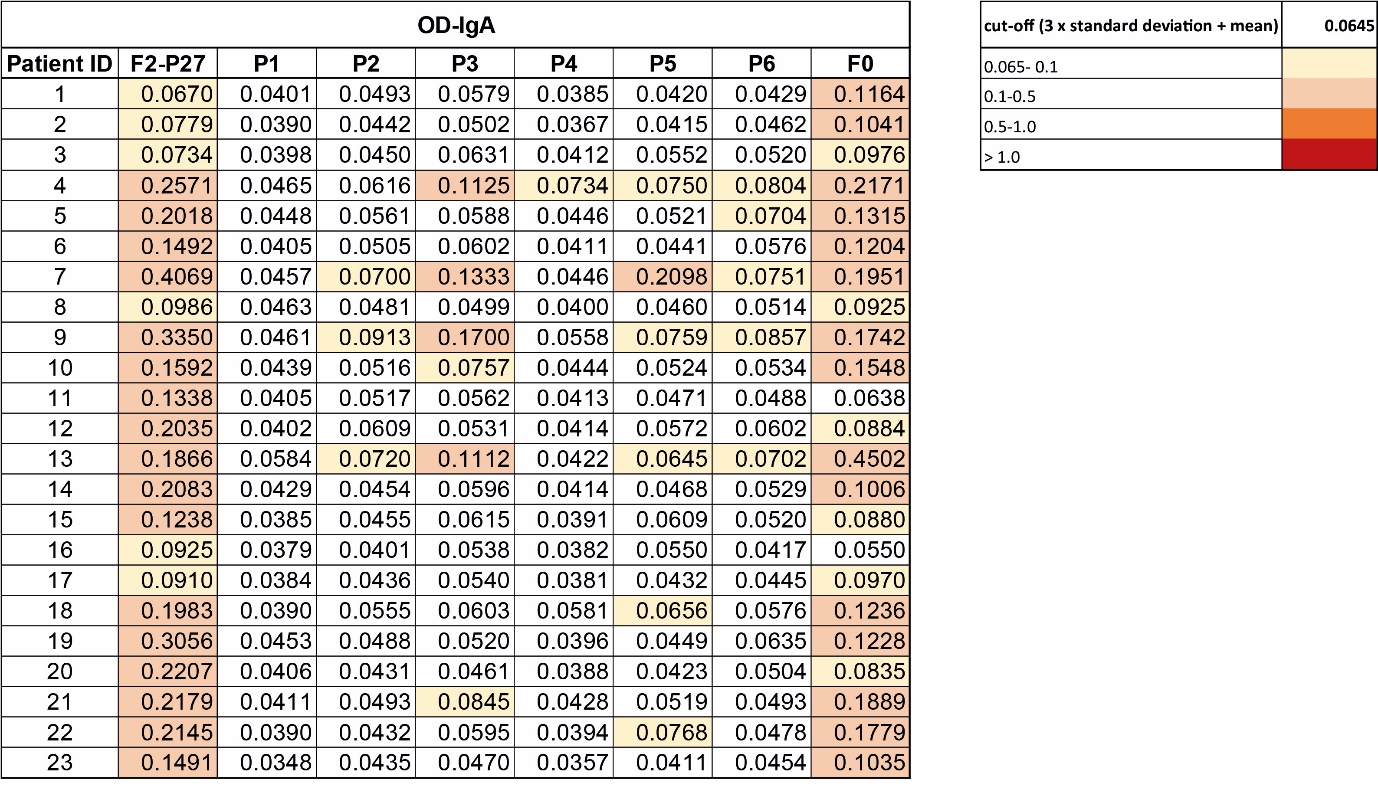


Supplemental Figure 2


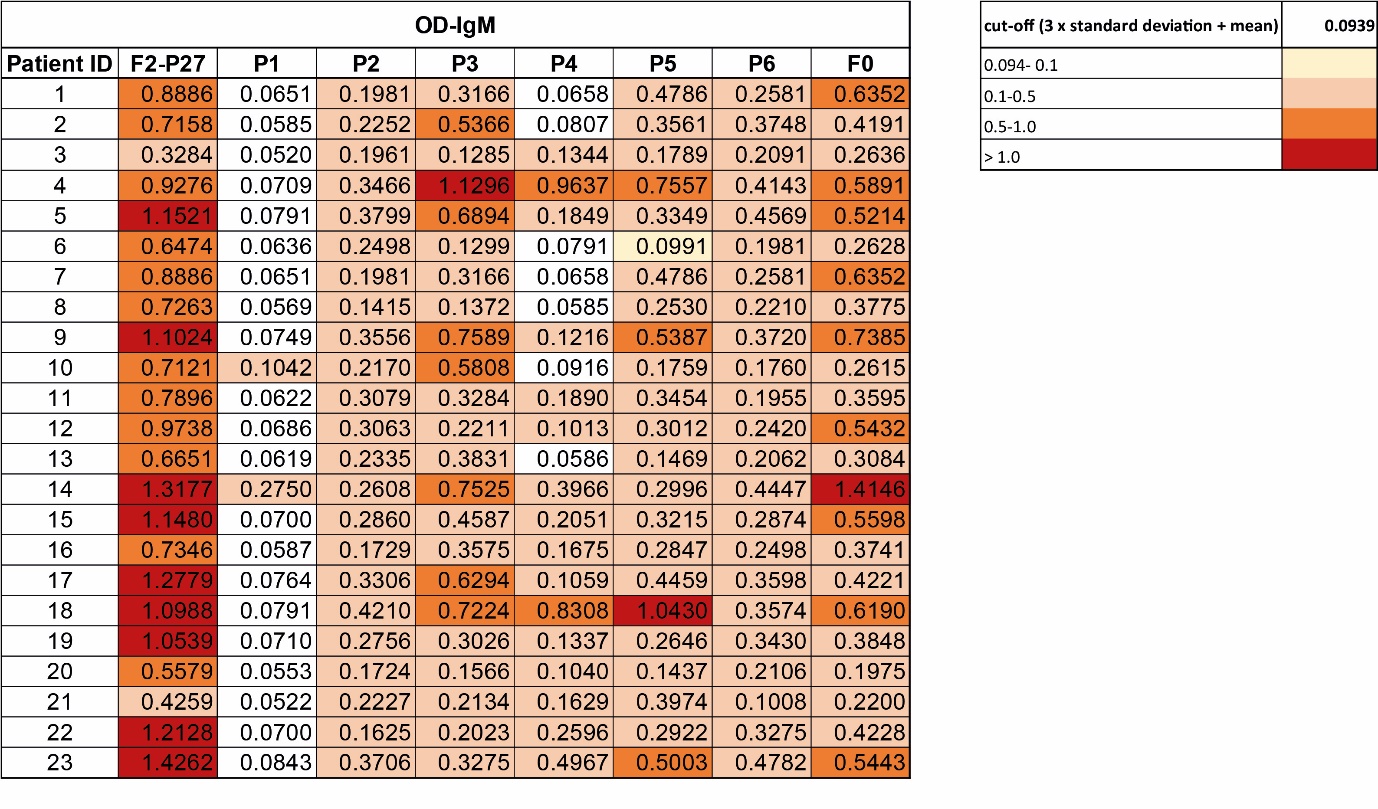


Supplemental Figure 3


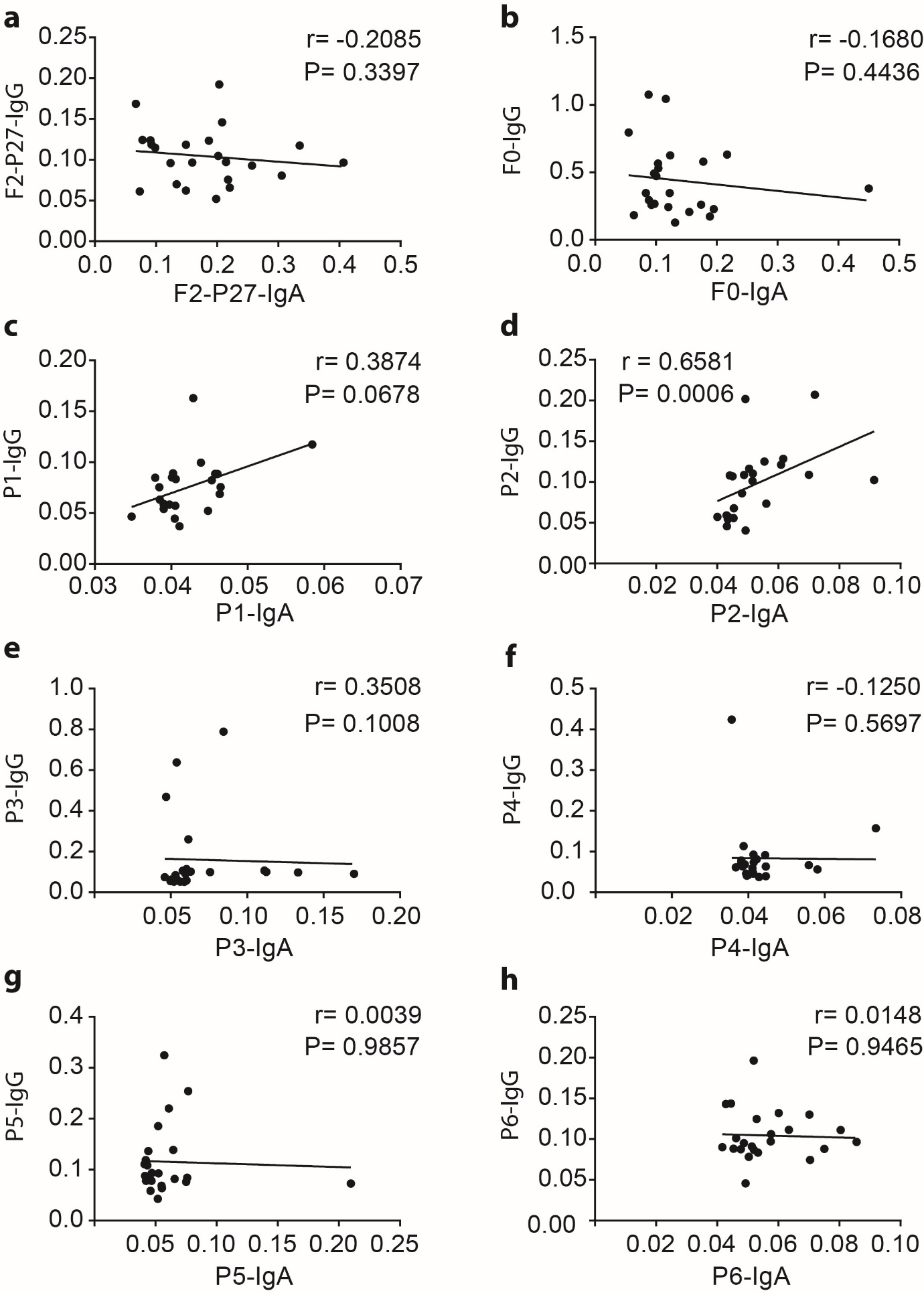


Supplemental Figure 4


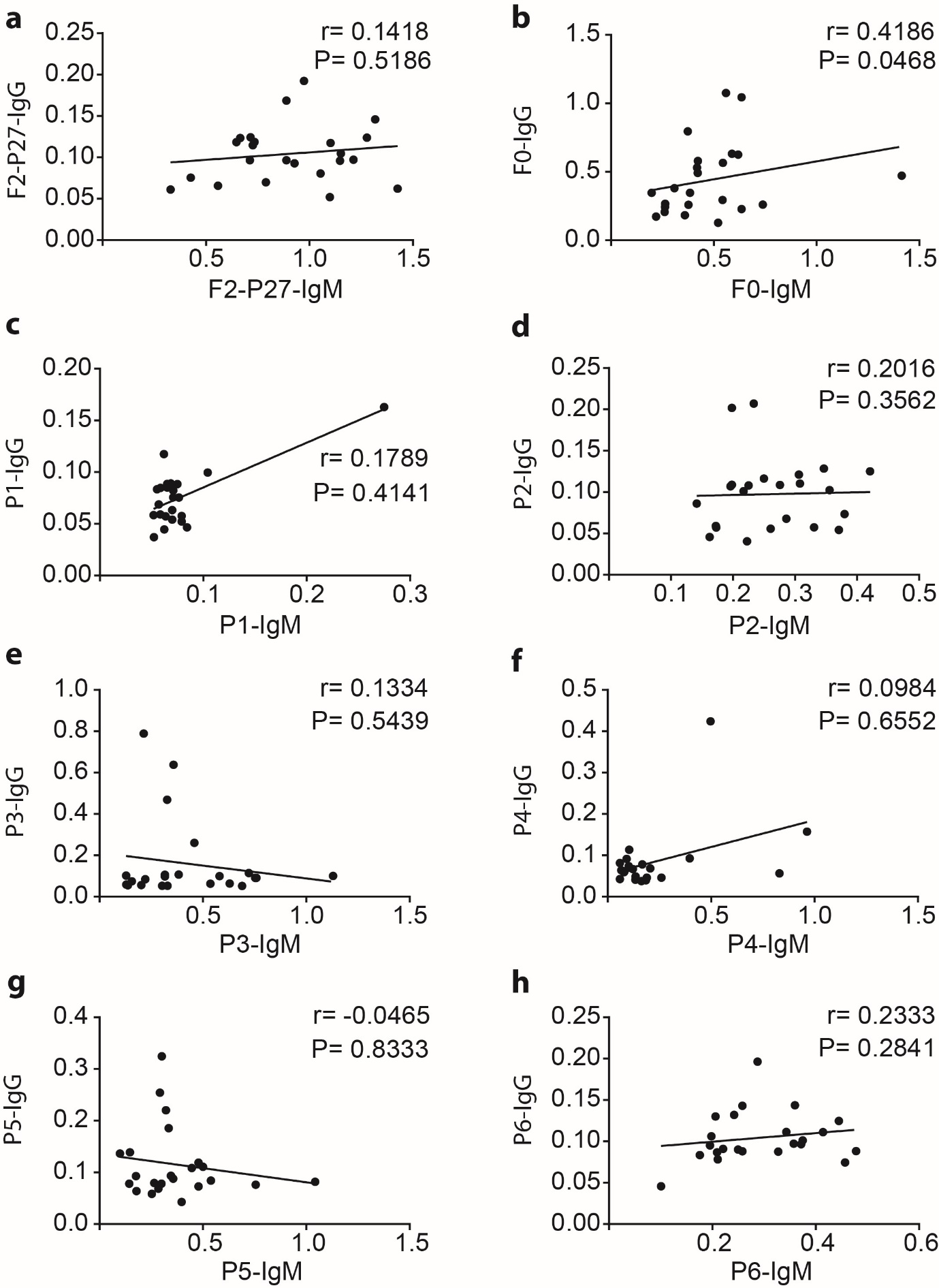


Supplemental Figure 5


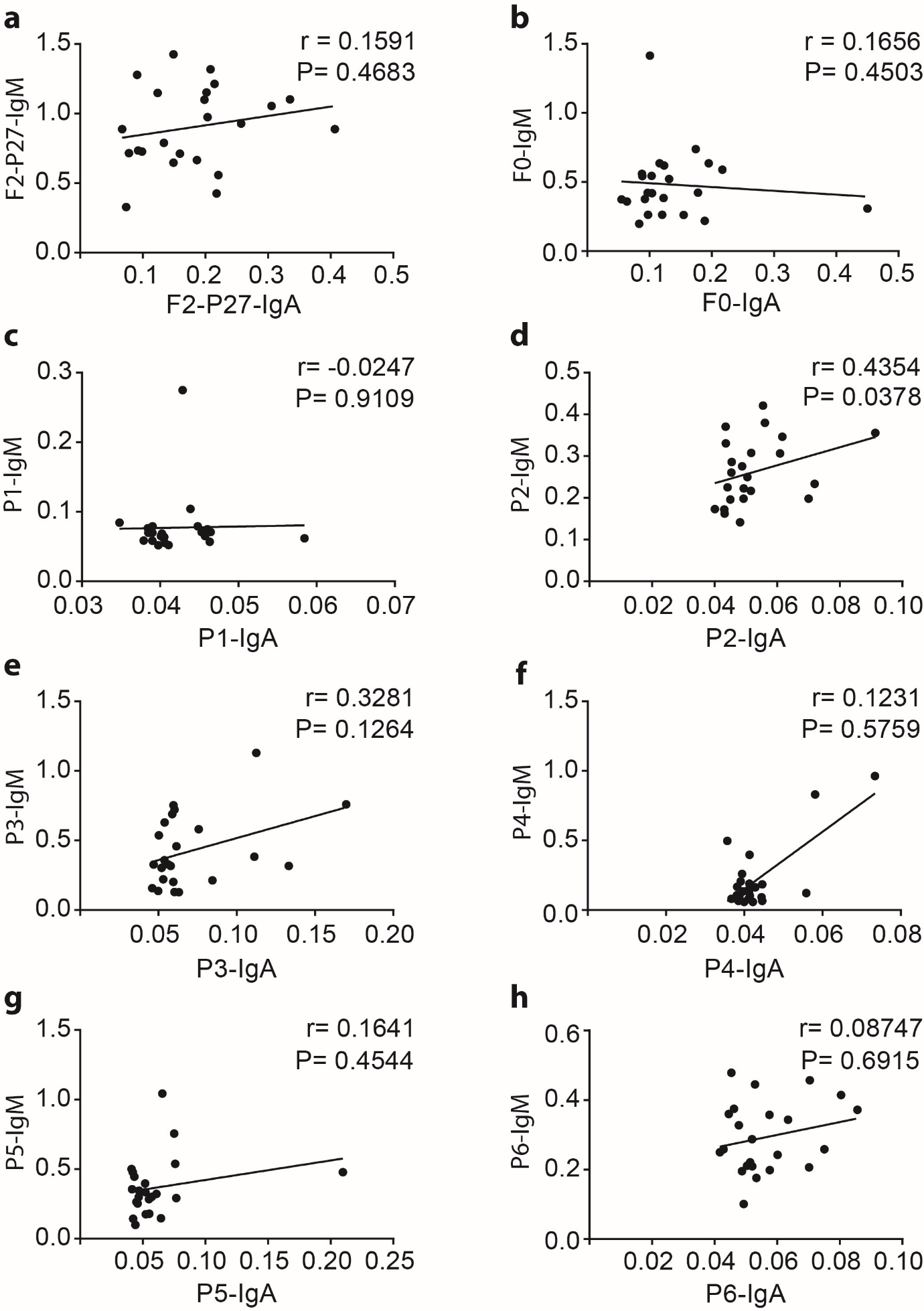


Supplemental Figure 6

**Original Pictures:**

*Recombinant F2-P27 protein*

Depicted are two Coomassie-stained SDS-PAGEs. Upper gel shows F2-P27 protein purification. Lower gel shows pure protein samples obtained during Ni-NTA affinity protein purification. The lower gel was used for the Figure 2 preparation, displayed in the manuscript. Therefore, the third protein band (lower gel) and marker lane, were cropped using Photoshop and further figure adjustments (labeling and outline) were performed by using Adobe Illustrator CS5 software.


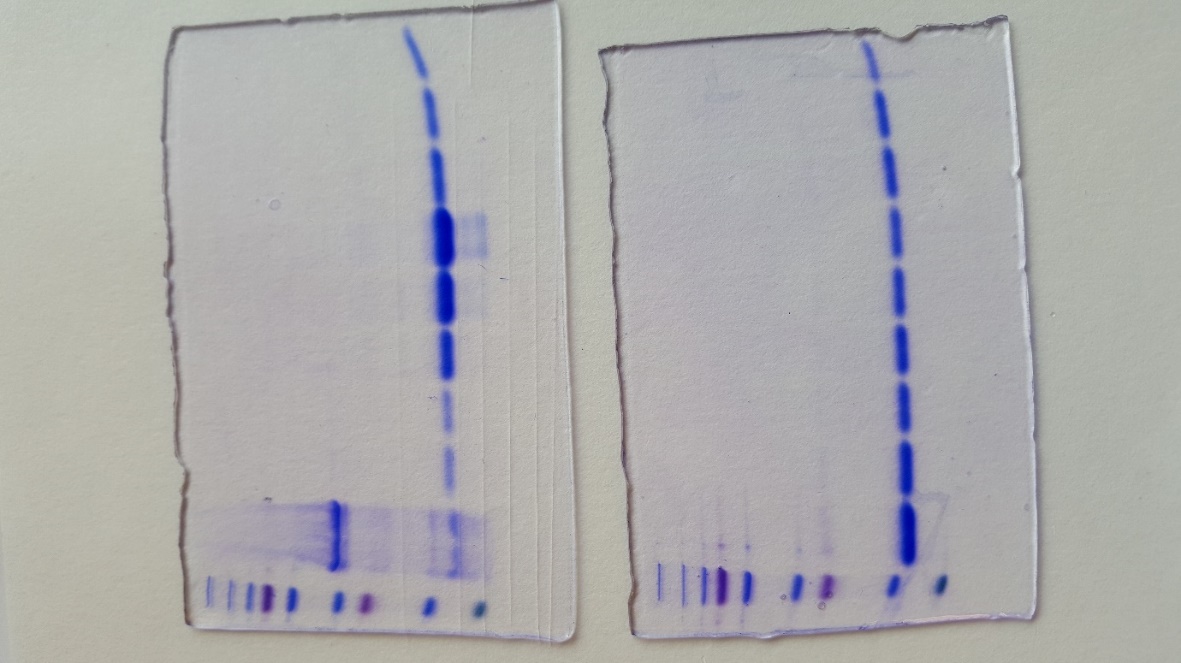


*Recombinant F0-protein*

Depicted is one Coomassie-stained SDS-PAGE, which shows F0 protein samples obtained during Ni-NTA affinity protein purification. This gel-image was used for the preparation of Figure 4a, displayed in the manuscript. Therefore, the third protein band and the marker lane, were cropped using Photoshop and further figure adjustments (labeling and outline) were performed by using Adobe Illustrator CS5 software.


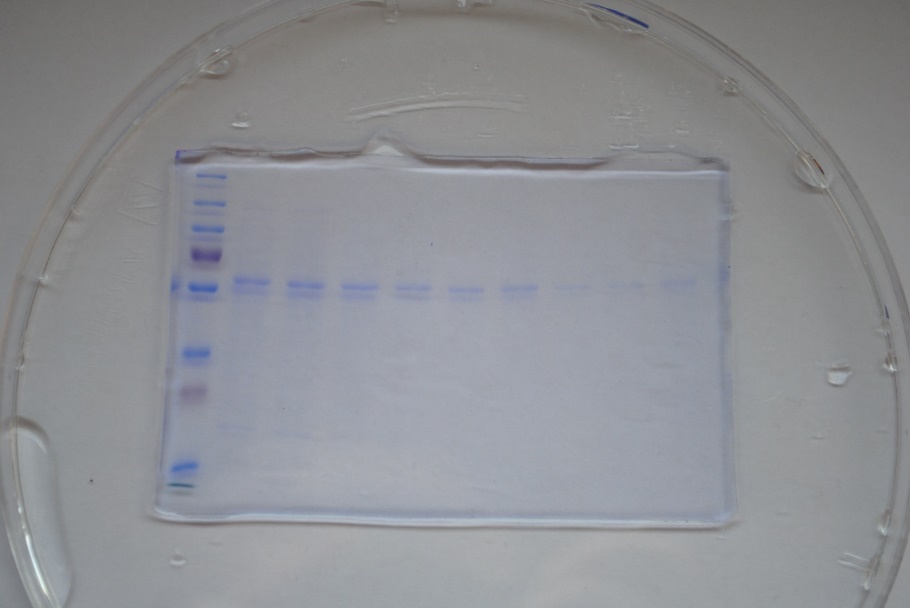

Supplement: Supplementary file 1 — Supplementary Information. [file 41598_2021_82893_MOESM1_ESM.docx]
